# Telework and Psychological Health in Hospital Staff during the First Wave of the COVID-19 Epidemic in France

**DOI:** 10.3390/ijerph181910433

**Published:** 2021-10-03

**Authors:** Carole Pelissier, Joelane Paredes, Martine Moulin, Thierry Bitot, Eric Fakra, Luc Fontana

**Affiliations:** Hospital University Center of Saint-Etienne, Université Lyon 1, Université de St Etienne, IFSTTAR, UMRESTTE, UMR_T9405, 42005 Saint-Etienne, France; p.joelane@hotmail.fr (J.P.); martine.moulin@chu-st-etienne.fr (M.M.); Thierry.bitot@chu-st-etienne.fr (T.B.); Eric.Fakra@chu-st-etienne.fr (E.F.); luc.fontana@chu-st-etienne.fr (L.F.)

**Keywords:** telework, hospital workers, mental health, stress, COVID-19 pandemic

## Abstract

Background: The COVID-19 pandemic led to a change in work organization with the development of telework. The purpose of this study was to assess the prevalence of anxiety and depressive symptoms in teleworking staff in a university hospital center in France during the first lockdown, and to identify personal, medical and occupational factors associated with anxiety disorder. Methods: A cross-sectional observational study was conducted in 474 hospital staff working from home during the first lockdown. The sociodemographic, occupational and medical information (anxiety and depressive disorders measured on the Hospital Anxiety and Depression (HAD) scale) was collected by an anonymous online self-administered questionnaire. The variables associated with anxiety disorder were investigated by a univariate analysis (chi² and Fisher tests) and a multivariate analysis (logistic regression model). Results: Three hundred and forty hospital staff participated in the study (72% response rate). Of the participants, 106 subjects (32.1%) showed signs of an anxiety disorder and 26 (7.65%) of a depressive disorder. An anxiety disorder was significantly associated with mental workload, changes in working hours, difficulties in teleworking due to issues of internet connection or due to noise, difficulties in combining family and occupational life, sleep disturbance, worry about media information and worry about the health of a loved one. An anxiety disorder remained associated with occupational stress and personal stress during lockdown after a multivariate logistic regression. Conclusions: This study highlighted the association between an anxiety disorder and perceived occupational and personal stress levels in hospital staff teleworking during the first lockdown. Stress management workshops could be proposed to hospital staff. Prevention of anxiety requires reinforced medical monitoring and reduced stress.

## 1. Introduction

Telework refers to any form of work organization in which work that could have been performed on the employer’s premises is accomplished elsewhere by an employee on a voluntary and planned basis using information and communication technologies [1]. In recent years, a few studies have explored the impact of telework on workers’ health and experience of working conditions. According to Lasfargue et al., telework is associated with longer working time, increased perceived workload and better quality of personal life, with less fatigue and stress [2]. A 2016 U.S. study of telecommuting intensity showed that its health benefits followed an inverted-U, moderate telecommuting providing greater benefit than very low or very high intensity [3]. In France, the legal framework of teleworking has been enshrined in the Labor Code since 2012, and the status and rights of the teleworker as well as the conditions for setting up telework in an establishment are defined therein [4]. Adopting new technology requires organizational change and individual adaptation [5]. The implementation of telework requires preparation such as: identification of possible telework tasks, acquisition of specific software, computer equipment adapted to the employee’s home and facilitation of internet connections.

The COVID-19 health crisis and lockdown led to a sudden increase in telework for many employees. COVID-19 is a contagious human-to-human infectious disease caused by a coronavirus, SARS-CoV-2, to which the majority of the population was not immune [6]. Since the beginning of 2020, SARS-CoV-2 has spread to several continents and is responsible for a large number of deaths [7,8]. In France between 1 March and 18 May 2020, there were 98,853 confirmed cases with a hospital admission, and 27,834 deaths. To reduce the risk of person-to-person viral transmission during the COVID-19 pandemic, the French government introduced various measures, including a lockdown from 17 March 2020 to 11 May 2020, with social distancing and self-isolation strategies, and implemented numerous measures, including quarantine, reducing the use of public transport and temporarily canceling work and school, to control this disease. People were only allowed to leave their homes for proven unavoidable reasons, such as health matters, basic necessities and work for those who could not work from home [9]. The lockdown forced many employees to telework with their families at home. The need to telework from home in the presence of one’s family in the context of a health crisis may have been accompanied by increased stress and the onset of anxiety disorders.

A recent review investigated the relationships between telework and health. The authors identified benefits (stress reduction, greater flexibility, better work–life balance/control) and health problems (musculoskeletal problems, psychological problems) [10]. The health and occupational uncertainty that pertains to the epidemic crisis context is suspected to have been an important source of personal stress, as well as the collision of personal and work lives [11].

The COVID-19 pandemic has profoundly changed the working conditions of hospital staff (increased mental, emotional and physical workload, changes in work organization with the implementation of sudden telecommuting), which makes them most vulnerable to anxiety disorders [12,13].

Our research hypothesis is that telework during lockdown in a health crisis is associated with an increased prevalence of anxiety symptoms among workers.

The objectives of the present study were to evaluate the prevalence of anxiety and depressive symptoms in hospital staff teleworking in the context of a lockdown, and to investigate the associated medical, personal and occupational factors with anxiety symptoms.

## 2. Methods

The study design consisted of a cross-sectional questionnaire survey.

### 2.1. Target Population

The data were collected from 26 May to 10 June 2020. The target population was hospital staff teleworking during the period from 17 March to 10 May 2020, which included: directors, administrative officers, executive assistants, health managers, computer technicians, psychologists, medical secretaries, social workers and other staff.

### 2.2. Study Sample

The hospital staff of the University Hospital of Saint-Etienne working from home during the study period were invited to respond voluntarily to a self-administered online survey, 15 days after the end of the lockdown.

Inclusion criteria of the eligible subjects:

- To be over 18 years old;
- To be employed by the University Hospital of Saint-Etienne;
- Have been teleworking for at least 1 month.

Exclusion criteria for the eligible subjects:

- Be off work or on leave at the time of inclusion in the study.

Of the hospital staff, 474 eligible employees (81% female, 19% male) including 8% paramedic staff were contacted by email. They received clear and comprehensible information on the study objectives and procedure, and were free to decline participation. The review board approval (IRBN722020/CHUSTE) was obtained before starting the study. If they agreed to participate in the study, they completed an anonymous online questionnaire via the LimeSurvey application.

### 2.3. Measurements

We developed a self-reported questionnaire to collect data on the demographic, occupational and medical characteristics. The self-administration time was approximately 10 min.

The French version of the Hospital Anxiety and Depression (HAD) scale has good reliability and discriminant validity: the internal consistency of the two scales is good (concerning the anxiety scale, Cronbach’s is alpha 0.81, and concerning the depression scale, Cronbach’s is alpha 0.78) [14]. The main endpoint (anxiety symptoms) was assessed on the validated French version of the Hospital Anxiety and Depression (HAD) scale. The “anxiety” dimensions were rated on 3 levels: no symptoms (score ≤ 7), doubtful (8–10) and certain (≥11). A cut-off at 8 points defined the clinical signs suggestive of anxiety disorder.

The anonymous self-administered questionnaire covered 3 areas with single- and multiple-choice questions.

*Personal:* gender, age, number of children in the household, type of accommodation.

*Occupational:* occupational category, working hours, weekly frequency of telework, change in working hours, location of telework, difficulties experienced in teleworking, lack of communication with colleagues and with hierarchy, self-estimated level of exposure to COVID-19 and increase in workload. The perceived stress related to personal and occupational life before and during the lockdown was assessed on a visual analogue scale (VAS) [15]. A cut-off at 7 points defined the clinical signs suggestive of stress. The participants were asked about their experience of their working conditions during the lockdown.

*Medical:* history of anxiety disorder, psychotropic treatment, psychological therapy, changes in frequency of physical activity, alcohol consumption and smoking, quality of sleep, SARS-CoV-2 infection and worry related to risk of infection, personal health status, a loved one’s health status, work conditions, information transmitted by the media and end of the lockdown.

### 2.4. Analysis

A descriptive analysis was made of the sample’s sociodemographic, occupational and medical characteristics.

A univariate analysis assessed the association between anxiety symptoms and sociodemographic, occupational and medical factors. Chi² and Fisher tests were applied as appropriate. The significance threshold was set at 5%. Variables significantly associated with anxiety disorder were introduced in a stepwise logistic regression model. Variables with *p*-value ≤ 0.1 were included in the multivariate model on a descending procedure, and variables with *p*-value < 0.05 were kept in the model. The analyses used SAS 9.4 software.

## 3. Results

### 3.1. Sociodemographic, Occupational and Medical Characteristics

As shown in Table 1, of the 474 eligible hospital staff, 340 (76% female, 24% male) responded: response rate, 72%. More than half of the respondents were over 50 years of age. Twenty percent lived with a child under the age of seven and more than a third were actively involved in their children’s schoolwork during the lockdown. A quarter of the respondents considered their exposure to COVID-19 at home to be average to very high. Almost two-thirds expressed concern about contracting COVID-19, but only four reported having actually contracted it. More than a third reported a deterioration in their sleep quality. During the lockdown, 17.7% of respondents increased their alcohol consumption, 11.8% increased their smoking and more than a third reduced their physical activity. More than three-quarters of the sample was made up of non-healthcare personnel. There was an increase in the prevalence of perceived high stress in personal and occupational settings. More than a third of the respondents reported an increased workload during the lockdown. The majority had not done telework before the lockdown, but more than half teleworked 5 or more days per week during the lockdown. More than two-thirds did not report any communication difficulties with colleagues or management.

### 3.2. Prevalence of Anxiety and Depression Symptoms

As shown in Table 2, Of the participants, 106 respondents (31%) presented anxiety symptoms and 26 (7%) presented depressive symptoms.

### 3.3. Relations between Anxiety Symptoms and Occupational/Personal Factors on Univariate Analysis

As shown in Table 2, after a univariate analysis, anxiety symptoms were significantly associated (*p*-value < 0.05) with:

- Personal and occupational stress level pre-lockdown (respectively OR = 2.26 (1.52–2.35); OR = 1.70 (1.21–2.39)) and during the lockdown (respectively OR = 3.32 (2.57–4.30); OR = 2.42 (1.78–3.30));
- Mental workload (OR = 1.6 (1.10–2.33));
- Changes in working hours for occupational reasons (OR = 1.58 (1.16–2.16));
- Difficulties in teleworking due to an unreliable internet connection (OR = 1.59 (1.15–2.18)) or noise (OR = 2.42 (1.81–3.25));
- Difficulties in combining family and occupational life (OR = 1.37 (1.00–1.86)).

In contrast, anxiety symptoms were not significantly associated with self-estimated occupational exposure to COVID-19 or the COVID-19 status of the department in which the respondent was working.

### 3.4. Relations between Anxiety Disorder and Medical Factors on Univariate Analysis

As shown in Table 2, after a univariate analysis, anxiety symptoms were associated (*p*-value < 0.05) with:

sleep disturbance (OR = 1.43 (0.90–2.26)) and
worry about information in the media (OR = 1.69 (1.18–2.43)),
about the health of a loved one (OR = 1.77 (1.30–2.40)) and
about the implementation of the end of lockdown (OR = 1.46 (1.03–2.06)).

In contrast, anxiety symptoms were not significantly associated with a change in alcohol consumption, in smoking habits or in physical activities.

### 3.5. Relations between Anxiety Symptoms and Occupational and Medical Factors on Multivariate Analysis

As shown in Table 2, a multivariate logistic regression showed that anxiety disorder remained associated with occupational stress (OR = 1.55 (1.08–2.22)) and personal stress (OR = 2.10 (1.53–2.89)) during the lockdown.

## 4. Discussion

Our study highlights the prevalence of anxiety symptoms among hospital staff (mostly non-caregiver) who were forced to telework in the context of increased professional and personal stress levels associated with the COVID-19 pandemic lockdown. This study underlines the association between anxiety symptoms and the increase in mental workload, the difficulties in teleworking related to family–work balance, noise and internet connection during the lockdown.

The prevalence of anxiety disorder and depressive symptoms in teleworking staff was 31% and 7%, respectively. The prevalence of anxiety in the present study was consistent with that reported by Carrion et al., who showed that 33.6% of teleworking health professionals had anxiety disorders, but the prevalence of depression was lower (7% versus 27%) [16], perhaps because our target population was probably exposed to a lower emotional load because they were teleworking during the COVID-19 pandemic. In a cross-sectional population-based online survey conducted from 28 February 2020, to 11 March 2020 in China, the authors reported a 31.6% rate of anxiety (95% CI, 31.2–32.0%) [17].

Telework, also known as remote working, is gaining popularity and becoming a common feature in the economy, due not only to advances in digital technology but also to changing attitudes about where and when work should be performed and how performance should be measured [18]. As the lockdown began, working from home was required by all of those who could reasonably be expected to do so, and offices and other workplaces were closed down [19]. The majority of the respondents had not used teleworking before the lockdown, but more than half teleworked 5 or more days per week during lockdown. More than half reported sharing their workspace with others in the household. The study highlighted an increase in the rate of high stress levels during the lockdown in a changing environment. These results are similar to those of Carrion and Anderson, who reported difficulties in finding suitable space for working, in access to equipment and in the reliability of an internet connection [16,19]. Almost half of the respondents, who were mainly female, had to help their children with schoolwork, and more than a third reported difficulty in combining family and working life. These results are consistent with those of Deirdre et al., who showed that many parents had to juggle work commitments with the increased demands on their time, including the practical aspects of supervising children’s learning, exercise and play [19]. A study of the general Spanish population during the COVID-19 pandemic showed that anxiety was positively associated with the female gender and with the time spent helping their children, as in our study [20]. Our study also found that anxiety was associated with annoyance by noise, as highlighted in the study by Amerio et al. [21]. Noise can interfere with concentration and make telecommuting more difficult.

Previous research showed that anxiety and a depressive mood were associated with smoking and alcohol abuse, as both are used to cope with stress [22,23]. In the present study, 11.8% of respondents reported an increase in smoking and 17.7% reported an increase in alcohol consumption since the lockdown. The increase in smoking was lower than for Guignard et al., with 26.7% of respondents in a sample of the general French population interviewed during the same period [24]. This difference could be related to the high workload of the present respondents, who had little time for smoking breaks. On the other hand, the prevalence of increased alcohol consumption was higher than for Guignard et al. (17.7% versus 10.7%) but similar to that in the study by Jacob et al. [25]. Most studies reported decreases in physical activity during the lockdown [26]. In the present study, 42.2% of the respondents reported decreased physical activity during the lockdown, compared to 52.8% in the French NutriNet-Santé cohort study of 37,252 French adults who filled out lockdown-specific questionnaires in April–May 2020 [27]. The decrease in physical activity may be due to the closure of gyms during the lockdown and travel restrictions. The present study found no significant association between a change in alcohol consumption, smoking or physical activities and the prevalence of anxiety disorder. The prevalence of poor sleepers in the present population (35.8%) was consistent with the 36.38% rate for the general population in China during the epidemic in an online survey from 18 to 25 February [28]. Our results showed an association between poor sleep quality and anxiety, consistent with the literature. Frontini et al. found that individuals who reported being satisfied with the quality of their sleep during lockdown had lower levels of anxiety [29]. According to Franceschini et al., all of the highlighted COVID-19 stressors seemed to trigger elevated cognitive and physiological hyperarousal, in a vicious circle that may have impaired sleep quality [30]; those who had high levels of stress, anxiety and depression also had a higher probability of a sleep disorder [30]. The lockdown is characterized by self-isolation, social distancing, loss of freedom and negative emotions, such as fear, which may lead to anxiety.

The present study in teleworking hospital staff showed that anxiety symptoms were significantly associated with the personal or occupational stress level. Our findings were consistent with those of Rodriguez et al., who reported that the pandemic induced moderate to severe levels of anxiety at work and at home in emergency physicians [31]. In the present study, the respondents reported worry for their family. This was consistent with the cross-sectional observational study of doctors, nurses and other hospital staff throughout Hunan province between January and March 2020 by Cai et al., who found that the main factors associated with stress were worry for personal safety, worry for family and worry for patient mortality [32]. On the other hand, in the present study, anxiety symptoms were not significantly associated with self-estimated occupational exposure to COVID-19 or the COVID-19 status of the department in which the respondent was working. This result could be explained by the fact that telecommuting reduces exposure to SARS-CoV2 within wards.

The worldwide coronavirus outbreak has put hospital staff under stress [33], and led to an increase in the workload for healthcare and other hospital workers due to organizational changes in the hospitals. More than two-thirds of respondents reported an increase in their workload, and more than half reported feeling overworked. There was an increase in the prevalence of high levels of personal or work-related stress during the lockdown. Geoffroy et al. presented a psychological support system for all of the hospital workers in Paris, France, during the COVID-19 epidemic [33]. They found that all of the hospital professions and departments had workers who were experiencing psychological distress, including non-frontline workers. They underlined “work-related stress”, with numerous changes at work, loss of routine and new procedures and materials [33]. Anxiety symptoms were the first cause for hospital workers to call the dedicated hotline. These results are consistent with those of the present study that highlighted an association between anxiety and increased personal and occupational stress levels.

Our study assessed the impact on the psychological health of the sudden implementation of telework among hospital staff in a health crisis context. This experience should be taken into account in order to better integrate the implementation of telework in business continuity plans to reduce, in particular, the exposure to stress of the hospital staff [11]. This study highlights the importance of taking into account the articulation with family life and the adequacy of the work environment at home (noise and poor quality of the internet connection) in the implementation of telework.

Some possible study limitations should be borne in mind. Firstly, the study was cross-sectional, and thus it was impossible to draw any conclusion about causal relations. Secondly, the sample size was small; however, the response rate was 72%. Thirdly, the anxiety and depressive symptoms were identified not on clinical examination but on a validated anxiety and depression scale. On the other hand, the study has the interest of evaluating the prevalence of anxiety and depression symptoms in hospital staff (mostly non-healthcare workers) working at home during the first period of the lockdown in France. An anxiety disorder was significantly associated with the personal or occupational stress level, mental workload, changes in working hours, difficulties in teleworking due to internet connection issues or to noise, difficulties in combining family and working life, sleep disturbance, worries about the media and worry about the health of a loved one. The multivariate logistic regression showed that an anxiety disorder remained associated with occupational stress and personal stress during the lockdown.

## 5. Conclusions

The implementation of physical distancing to limit SARS-CoV-2 transmission led to a rapid increase in telework. The total lockdown led to an increase in the level of stress felt by hospital staff teleworking in the context of a high workload and high levels of activity related to family life. This study shows a high prevalence of anxiety symptoms among telecommuting hospital staff at the time of the lockdown and highlights the significant association between high stress and an increased workload with anxiety symptoms. Stress management workshops could be proposed to hospital staff in this context of a health crisis. The prevention of anxiety requires reinforced medical monitoring and reduced stress. The implementation of telework should be accompanied by organizational and technical support in order to reduce stress levels. Furthermore, establishing clear boundaries and expectations with respect to family and friends on the one hand, and work organization on the other should help to reduce interference between work and family life.

## Figures and Tables

**Table 1:** Description of personal and professional medical factors.

Personal Factor		*N*	%	Occupational Factors		*N*	%
**Gender (*N* = 330)**	**Male**	80	24.2	**Job (*N* = 301)**	**Administrative** **director** **Executive**	22	7.1
	**Female**	250	75.8		**administrative worker** **Medical secretary**	56	18.0
**Age group (*N* = 340)**	**<37 years**	83	24.4		**IT technician**	26	8.4
	**(37–46 years)**	85	25.0		**Social worker**	25	8.0
	**(46–57 years)**	133	31.6		**Health manager**	63	23.4
	**≥57 years**	39	25.6				
**Family situation (*N* = 330)**	**In couple**	244	73.9		**Other (including 9 paramedics staff, 36 technicians or engineers)**	109	35.0
	**Single**	48	14.6	**Working hours** **(*N* = 311)**	**<25 h/wk**	22	7.1
	**Widowed, separated, divorced**	38	11.5		**26–35 h/wk**	89	28.6
**Number of children at home (*N* = 330)**	**None**	67	20.3		**36–48 h/wk**	172	55.3
	**1**	51	15.5		**>48 h/wk**	28	9.0
	**2**	145	49.9	**Increase in working hours (*N* = 311)**	**No**	184	59.2
	**>2**	67	20.3		**Yes**	127	40.8
**Number of children under 7 at home (*N* = 330)**	**None**	263	79.9	**Days of teleworking pre-lockdown** **(*N* = 310)**	**0**	296	95.5
	**1**	34	10.3		**1**	5	1.6
	**2**	30	9.1		**2**	2	0.65
	**>2**	3	0.9		**3**	2	0.65
**Types of accommodation** **(*N* = 330)**	**Apartment**	99	30.0		**4**	2	0.65
	**House**	230	69.7		**5**	2	0.65
	**Other**	1	0.3		**>5**	1	0.32
**Help with schoolwork** **(*N* = 330)**	**No**	185	56.1	**Days of teleworking during lockdown** **(*N* = 310)**	**1**	31	10.0
	**Yes**	145	43.9		**2**	63	20.3
**Medical factors**		*N*	%		**3**	24	7.7
					**4**	33	10.6
**Psychiatric history (*N* = 289)**	**No**	284	98.3		**5**	129	41.6
	**Yes**	5	1.7		**>5**	30	9.7
**History of anxiety disorder** **(*N* = 289)**	**No**	240	83.0	**Increase in teleworking (*N* = 311)**	**No**	127	40.9
	**Yes**	49	17.0		**Yes**	184	59.2
**Psychotropic treatment** **(*N* = 289)**	**No**	273	94.5	**Communication problems with colleagues (*N* = 311)**	**No**	222	71.4
	**Yes**	16	5.5		**Yes**	89	28.6
**Psychological/psychiatric treatment (*N* = 289)**	**No**	279	96.5	**Communication problems with hierarchy (*N* = 311)**	**No**	225	72.3
	**Yes**	10	3.5		**Yes**	86	27.7
**Change in sleep quality** **(*N* = 289)**	**Clear or moderate improvement**	45	15.6	**Same working hours (*N* = 340)**	**Yes**	142	41.8
	**No change**	141	48.8		**No**	198	52.8
	**Clear or moderate deterioration**	103	35.6		**-Change for occupational reasons**	108	31.8
**Change in alcohol consumption (*N* = 289)**	**No consumption**	79	27.3		**-Change for personal reasons**	114	33.5
	**No change**	135	46.7	**Same break time** **(*N* = 340)**	**Yes**	102	30.0
	**Slight to clear decrease**	24	8.3		**No**	238	70.0
	**Slight to clear increase**	51	17.7		**-Change for occupational reasons**	138	40.6
**Change in smoking habits** **(*N* = 289)**	**Non-smoker**	228	78.9		**- Change for personal reasons**	96	28.2
	**No change**	22	7.6	**Dedicated** **workspace (*N* = 311)**	**No**	146	46.9
	**Decrease**	5	1.7		**Yes**	165	53.1
	**Increase**	34	11.8	**Workspace with others present** **(*N* = 310)**	**No**	133	42.9
**Change in physical activity (*N* = 289)**	**No physical activity**	24	8.3		**Yes**	177	57.1
	**No change**	43	14.9	**Difficulty** **teleworking (*N* = 340)**	**No**	134	39.4
	**Slight decrease**	45	15.6		**Yes**	206	60.6
	**Clear decrease**	77	26.6	**Difficulty due to internet connection** **(*N* = 340)**	**No**	262	77.1
	**Slight increase**	39	13.5		**Yes**	78	22.9
	**Clear increase**	61	21.1	**Difficulty due to home space** **(*N* = 340)**	**No**	294	86.5
**Pre-lockdown personal stress (*N* = 340)**	**Low**	325	95.6		**Yes**	46	13.5
	**High**	15	4.4	**Difficulty due to workspace (*N* = 340)**	**No**	267	78.5
**Personal stress during lockdown (*N* = 340)**	**Low**	291	85.7		**Yes**	73	21.5
	**High**	49	14.3	**Difficulty due to family life** **(*N* = 340)**	**No**	262	77.1
**Estimated COVID-19 exposure away from work (*N* = 289)**	**Very high**	8	2.8		**Yes**	78	22.9
	**Moderate**	69	23.9	**Difficulty due to noise** **(*N* = 340)**	**No**	298	87.7
	**Low**	137	47.4		**Yes**	42	12.3
	**Very low**	75	26.0	**Other difficulties** **(*N* = 340)**	**No**	274	80.6
**Contracted COVID-19** **(*N* = 289)**	**No**	285	98.6		**Yes**	66	19.4
	**Yes**	4	1.4	**Difficulty combining work and family life (*N* = 311)**	**No**	205	65.9
**Worry about COVID-19 risk (*N* = 330)**	**No**	206	60.6		**Yes**	106	34.1
	**Yes**	134	39.4	**Type of department during lockdown** **(*N* = 310)**	**COVID-19 +**	13	4.2
**Worry about a personal factor (*N* = 340)**	**No**	297	87.4		**Mixed**	81	26.1
	**Yes**	43	12.6		**COVID-19 −**	120	38.7
**Worry about a loved one’s health (*N* = 340)**	**No**	248	72.9		**No in-hospital work**	96	31.0
	**Yes**	92	27.1	**Pre-lockdown occupational stress** **(*N* = 340)**	**Low**	290	85.3
**Worry about working conditions (*N* = 340)**	**No**	308	90.6		**High**	50	14.7
	**Yes**	32	9.4	**Occupational stress during lockdown** **(*N* = 340)**	**Low**	227	66.8
**Worry concerning media (*N* = 340)**	**No**	299	87.9		**High**	113	33.2
	**Yes**	41	12.1	**Perceived overwork (*N* = 311)**	**No**	142	45.6
**Worry about end of lockdown (*N* = 340)**	**No**	278	81.8		**Yes**	169	54.4
	**Yes**	62	18.2	**Increased mental load (*N* = 310)**	**No**	106	34.2
					**Yes**	204	65.8

**Table 2:** Personal, occupational and medical factors associated with anxiety in staff teleworking during the lockdown.

Factors		Anxiety Symptoms							
		No *N* = 234 (68.8%)		Yes *N* = 106 (31.2%)					
		*N*	%	*N*	%	OR	CI	ORadj	CI
**Gender**	**Male**	63	78.7	17	21.2	1 *			
	**Female**	161	64.4	89	35.6	1.68	1.06–2.64	/	
**Help with schoolwork**	**No**	134	72.4	51	27.6	1 *			
	**Yes**	90	62.1	55	37.9	1.38	1.0–1.88		
**Days per week teleworking during lockdown)**	**1**	22	71.0	9	29.0	1 *		/	
	**2**	35	56.6	28	44.4	1.53	0.83–2.83		
	**3**	20	83.3	4	16.7	0.57	0.20–1.64		
	**4**	18	54.6	15	45.4	1.57	0.80–30.5		
	**5**	92	71.3	37	28.7	0.99	0.53–1.83		
	**>5**	17	56.7	13	43.3	1.49	0.75–2.96		
**Change in working hours for occupational reasons**	**No**	171	73.7	61	26.3	1 **		/	
	**Yes**	63	58.3	45	41.7	1.58	1.16–2.16		
**Change in break time for occupational reasons**	**No**	148	73.3	54	26.7	1 *			
	**Yes**	86	62.3	52	37.7	1.41	1.03–1.93	/	
**Workspace with others present**	**No**	95	71.4	38	28.6	1 ▯			
	**Yes**	109	61.6	68	38.4	1.34	0.97–1.86	/	
**Difficulty teleworking**	**No**	101	75.4	33	24.6	1 *			
	**Yes**	133	64.6	73	35.4	1.44	1.01–2.04	/	
**Difficulty with internet connection**	**No**	190	72.5	72	27.5	1 **			
	**Yes**	44	56.4	34	43.6	1.59	1.15–2.18	/	
**Difficulty with family life**	**No**	193	73.7	69	26.3	1 ***		/	
	**Yes**	41	52.6	37	47.4	1.80	1.32–2.45		
**Difficulty with noise**	**No**	219	73.5	79	26.5	1 ****			
	**Yes**	15	35.7	27	64.3	2.42	1.81–3.25	/	
**Difficulty combining work and family life**	**No**	143	69.8	62	30.2	1 *			
	**Yes**	62	58.5	44	41.5	1.37	1.00–1.86	/	
**Pre-lockdown occupational stress**	**Low**	208	71.7	82	28.3	1 **			
	**High**	26	52.0	24	48.0	1.70	1.21–2.39	/	
**Occupational stress during lockdown**	**Low**	179	78.9	48	21.1	1 ****		1	
	**High**	55	48.7	58	51.3	2.42	1.78–3.30	1.55	1.08–2.22
**Increased mental load**	**No**	80	75.5	26	24.5	1 **			/
	**Yes**	124	60.8	80	39.2	1.60	1.10–2.33		
**Problems of communication with hierarchy**	**No**	157	69.8	68	30.2	1 *			
	**Yes**	48	55.8	38	44.2	1.46	1.07–2.00		/
**Problems of communication with colleagues**	**No**	153	68.9	69	31.1	1 ▯			/
	**Yes**	52	58.4	37	41.6	1.34	0.98–1.83		
**Pre-lockdown personal stress**	**Low**	229	70.5	96	29.5	1 **			/
	**High**	5	33.3	10	66.7	2.26	1.52–3.35		
**Personal stress during lockdown**	**Low**	223	76.6	68	23.4	1 ****		1	
	**High**	11	22.4	38	77.6	3.32	2.57–4.30	2.10	1.53–2.89
**Worry about a loved one’s health**	**No**	184	74.2	64	25.8	1 ***			/
	**Yes**	50	54.4	42	45.6	1.77	1.30–2.40		
**Worry about media**	**No**	213	71.2	86	28.8	1 **			/
	**Yes**	21	51.2	20	48.8	1.69	1.18–2.43		
**Worry about end of lockdown**	**No**	196	71.2	80	28.8	1 *			/
	**Yes**	36	58.1	26	41.9	1.46	1.03–2.06		
**Change in sleep quality**	**Improved**	30	66.7	15	33.3	1 *			/
	**No change**	99	70.2	42	29.8	0.89	0.55–1.45		
	**Deteriorated**	54	52.4	49	47.6	1.43	0.90–2.26		

Adjustment on socio-occupational and medical factors with *p*-value < 0.1; ▯ *p*-value < 0.1; * *p*-value < 0.05; ** *p*-value < 0.01; *** *p*-value < 0.001; **** *p*-value < 0.0001; OR: odds ratio; CI: confidence interval.

## Data Availability

The data presented in this study are available on request from the corresponding author. The data are not publicly available due to confidentiality of participants.
